# Supplementary material for: Uncovering minimal pathways in melanoma initiation
Source: Nat Commun. 2025 Jun 26;16:5369. doi: 10.1038/s41467-025-60742-0 (PMC12202705; doi:10.1038/s41467-025-60742-0)
Supplement: Supplementary file 1 — Supplementary Information [file 41467_2025_60742_MOESM1_ESM.pdf]

Suppl. Figure 1

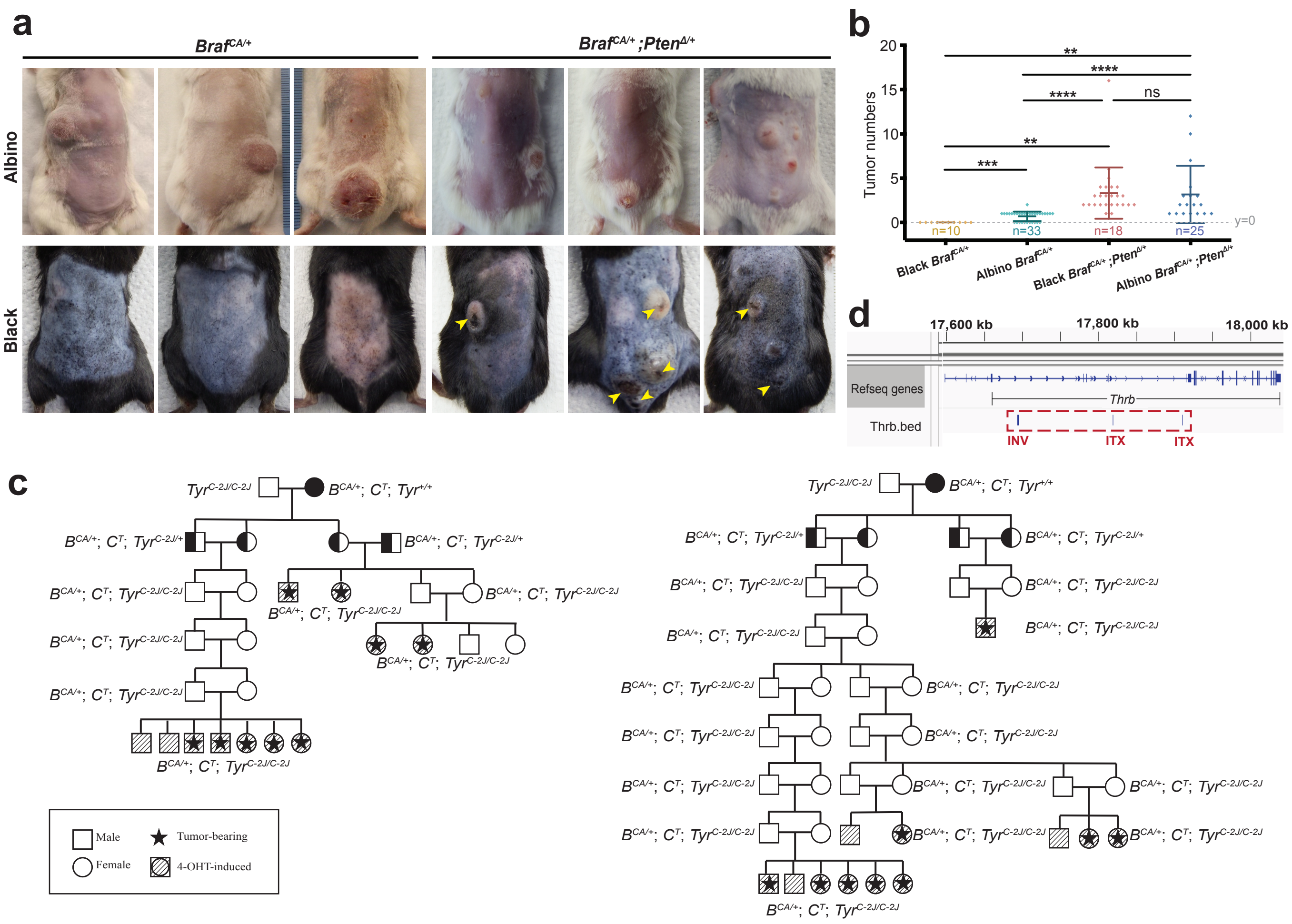

Supplementary Figure 1. A-B. (A) Additional representative images and (B) numbers of tumors per animal, of *Braf*$^{CA/+}$ and *Braf*$^{CA/+}$; *Pten*$^{\Delta/+}$ models in different coat-color backgrounds. Arrows in panel A highlight the scant pigment observed associated with tumors in black mice. Tumor numbers were compared using an unpaired two-tailed t-test. Asterisks denote statistical significance: $p < 0.05$ (*), $p < 0.01$ (**), $p < 0.001$ (***), $p < 0.0001$ (****); ns: not significant. Source data are provided as a Source Data file. **C.** Mouse family tree depicting the breeding scheme used to generate the Albino *Braf*$^{CA/+}$ mice. *B: Braf. C*^{T}*: Tyr::CreERT2.* Animals treated with tamoxifen were separated from littermates that were subsequently bred. **D.** Mapping of *Thrb* structural variants described in Fig. 1E. Note the variant types are inconsistent and the variant regions do not overlap. INV: Inversion. ITX: Intrachromosomal translocation. *Thrb* expression in the tumor cells of these samples is only barely detectable and is much lower than in other cell types (see Fig S3D).

Suppl. Figure 2

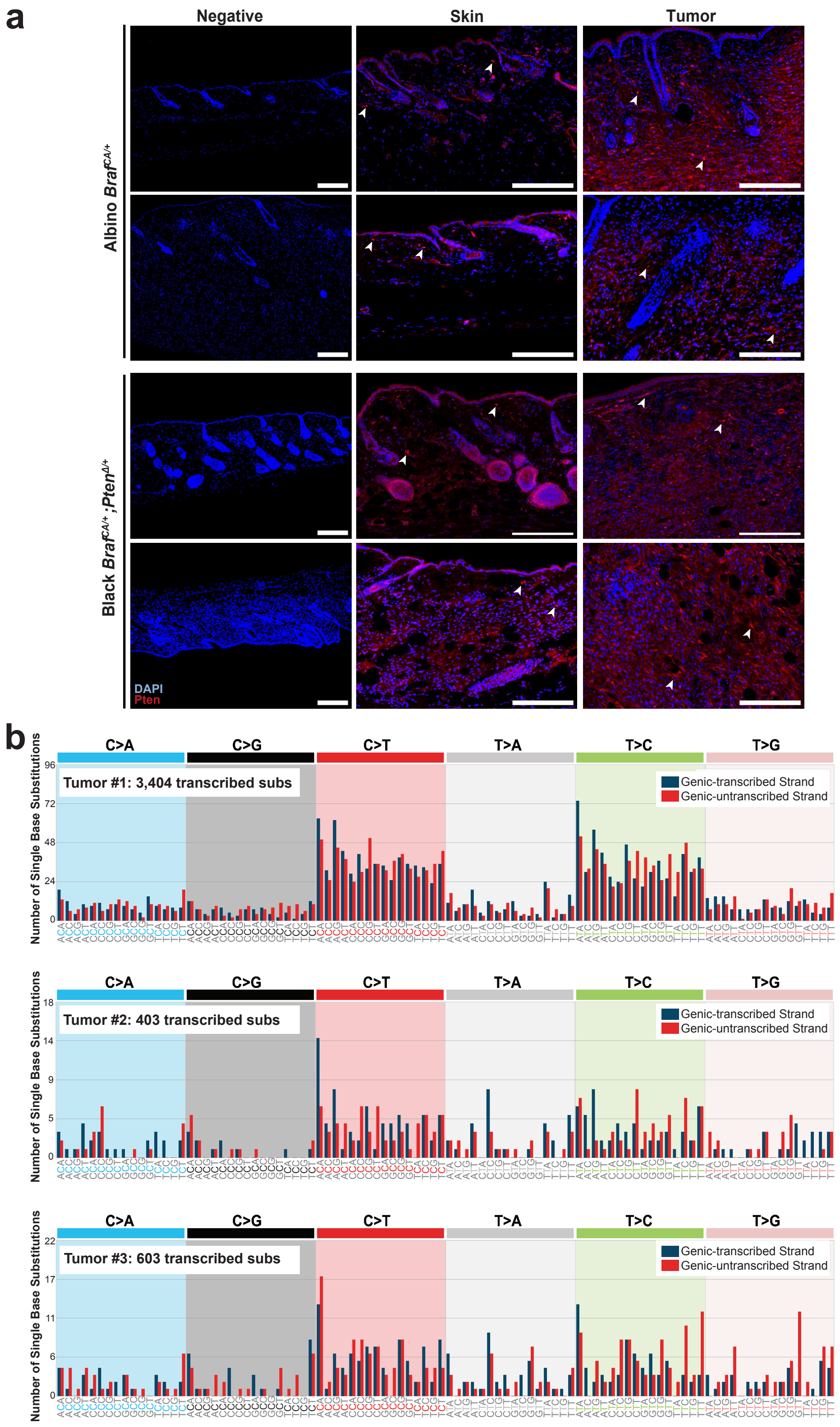

Supplementary Figure 2. A. Albino *Braf*$^{CA/+}$ and black *Braf*$^{CA/+}$; *Pten*$^{\Delta/+}$ tumors and adjacent skin were stained with a Pten antibody (red staining indicated by white arrows) and a DAPI nuclear stain. Two representative images from each tumor (representative of three different biological replicates) are shown. Scale bar: 200um. **B.** The number of single base substitutions from three Albino *Braf*$^{CA/+}$ tumors subjected to whole genome sequencing were analyzed by SigProfiler to identify mutational signatures. The trinucleotide contexts of each mutation are shown in the x-axis. Mutations are categorized by the transcribed or untranscribed strand. Subs: substitutions.

Suppl. Figure 3

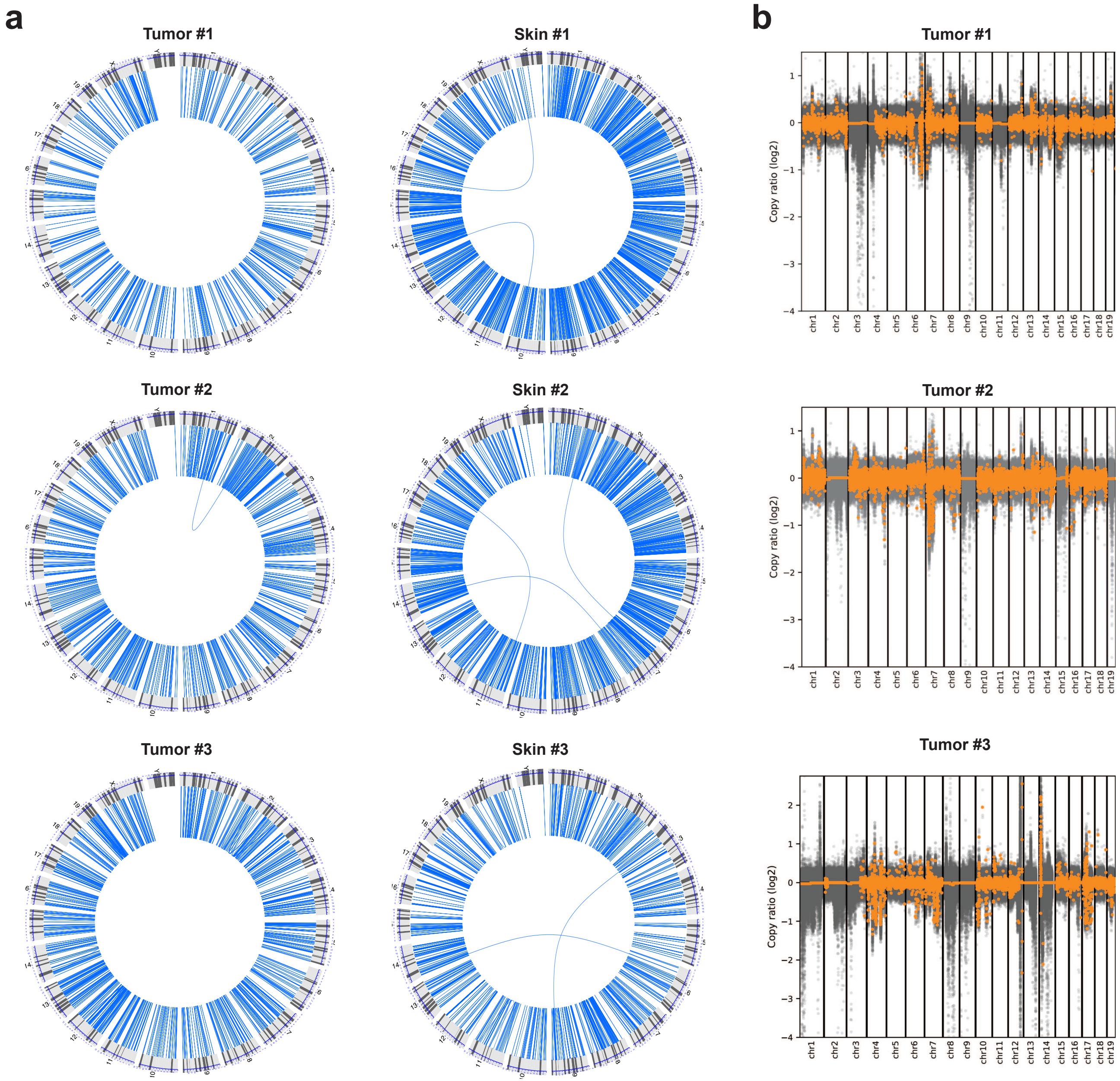

Supplementary Figure 3. A. Circos plots of structural variants identified by *BreakDancer* for the three albino *Braf*$^{CA/+}$ tumors subjected to WGS. Tumor sample structural variants were identified using both skin and spleen as control. Skin samples structural variants were identified using spleen as control. **B.** Whole genome profiles of log2 copy ratio by CNVkit for the three albino *Braf*$^{CA/+}$ tumors subjected to WGS. Grey dots denote bin level log coverages and orange dots denote segmentation calls by CNVkit.

Suppl. Figure 4

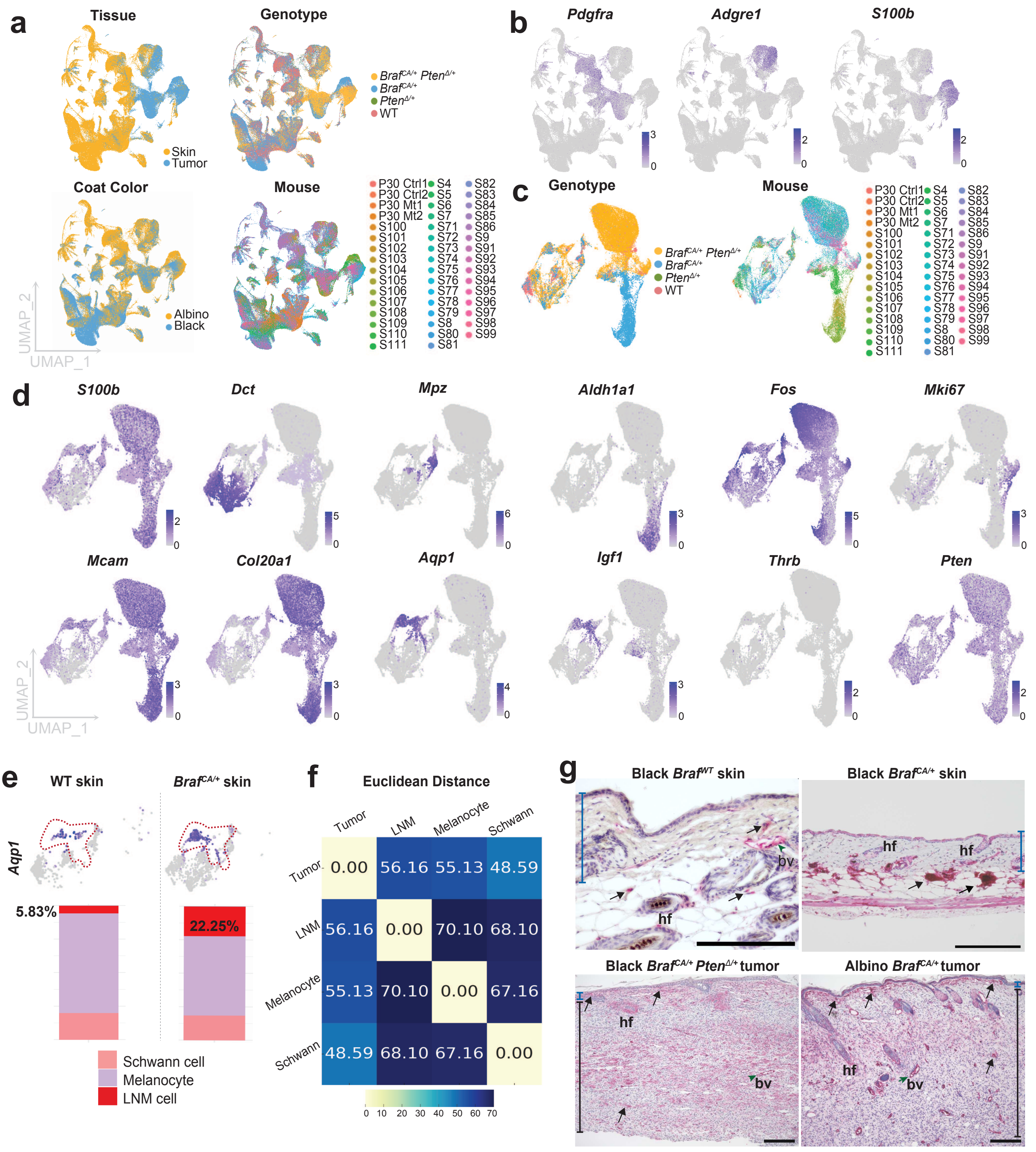

Supplementary Figure 4. A. Additional UMAP visualizations of cell distributions labeled by tissue, coat color, genotype, and mouse in the whole-skin single-cell RNA-sequencing data of 345,427 cells shown in Fig 2B. Detailed mouse metadata is included in Table S13. **B.** Feature plots highlighting cell-types of interest based on known marker genes: Fibroblasts (*Pdgfra*), Macrophages (*Adgre1*) and Principal tumor cells (*S100b*). Color bar denotes expression level. **C.** Additional UMAP visualizations labeled by genotype and mouse of the merged NC-derived clusters (35,527 cells from Fig 2C) **D.** Feature plots of the merged NC-derived clusters highlighting distinct cell-types. **E.** Feature plots of the LNM marker *Aqp1* and cell proportion plots in melanocyte cells across WT and *Braf*-mutant skin. Note the expansion of LNM cells in skin from black *Braf*$^{CA/+}$ mice (as tumors do not form in these mice, such cells cannot be tumor-derived). Color bar denotes expression level. **F.** Heatmap of Euclidean distance between NC-derived clusters using embeddings from the top 10 principal components (PCs). **G.** Immunohistochemistry of skin/tumor samples stained with an Aqp1 antibody that marks LNM cells (pink staining marks Aqp1+ cells; black arrows). Samples were counterstained with hematoxylin. Representative images of three independent biological replicates are shown. Note the strong staining in the nevi of skin samples. Aqp1 straining starts in the superficial dermis (blue bracket) and extends to the subcutis (black bracket) in tumors.hf: hair follicles. bv: blood vessels. Scale bar: 200um.

Suppl. Figure 5

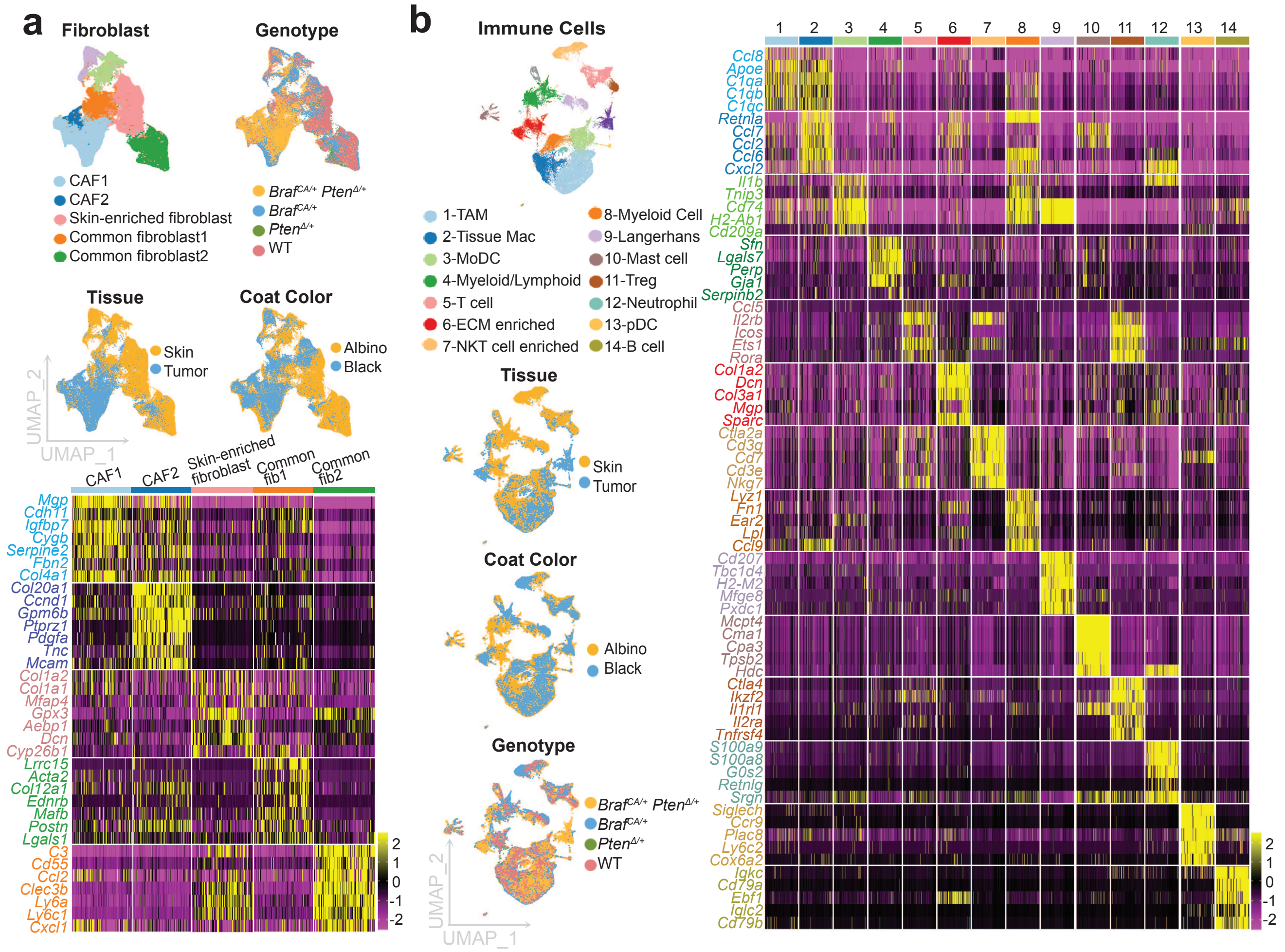

Supplementary Figure 5.A-B. Gene expression profiles of (A) 86,536 fibroblasts and (B) 51,747 immune cells associated with the skin and tumor microenvironments, as described in Fig 2B. Cells were subclustered, visualized by UMAP, and labeled by tissue of origin, coat color, and genotype. Each column in the heatmap represents a cell from the corresponding group. CAF: cancer-associated fibroblast. Fib: fibroblast. TAM: tumor-associated macrophage. MoDC: Monocyte-derived dendritic cell. ECM: Extracellular matrix. Treg: Regulatory T cell. pDC: Plasmacytoid dendritic cell.

Suppl. Figure 6

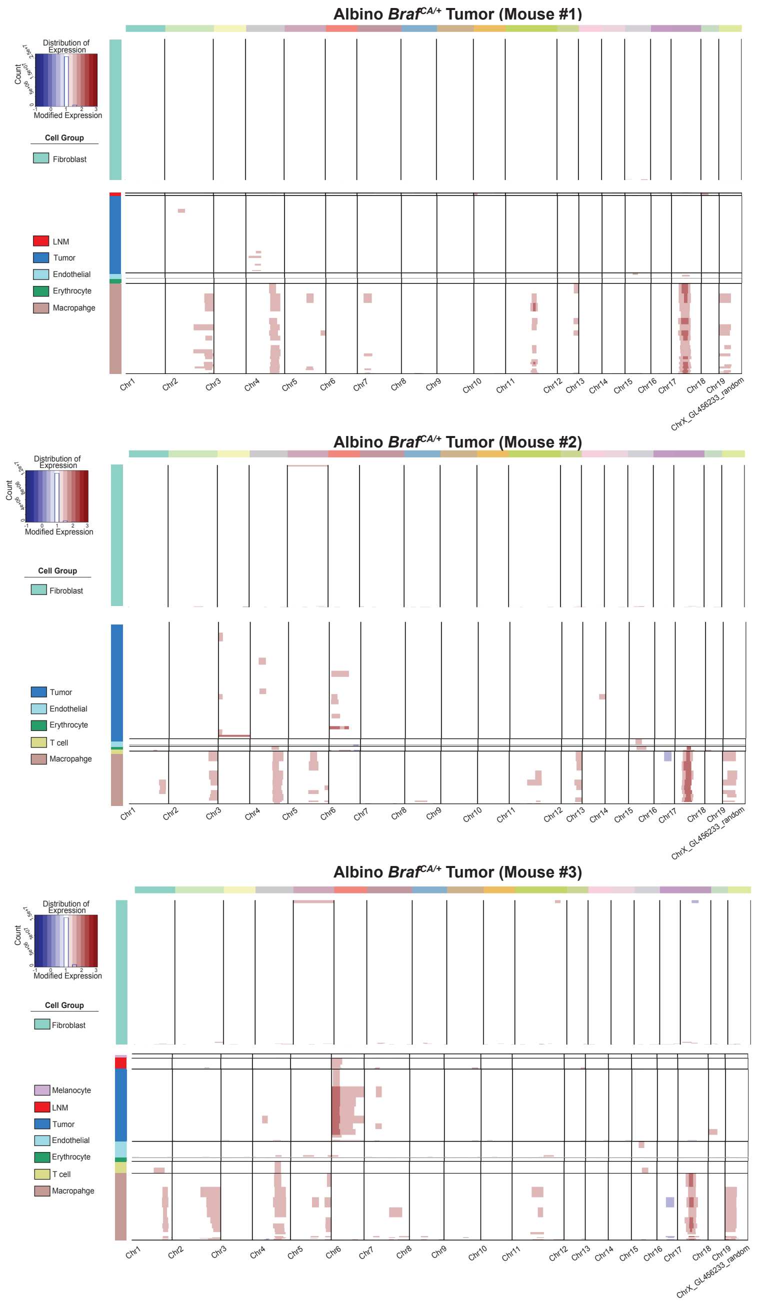

Supplementary Figure 6. Heatmap of inferred large-scale copy number variations (CNVs) from three Albino *Braf*$^{CA/+}$ mouse tumors. InferCNV was used to identify copy number variants from single-cell RNA expression profiles. Fibroblasts were used as a normal reference, and compared with melanocytes, LNM cells, tumor cells, endothelial cells, erythrocytes, T cells, and macrophages. X-axis and Y-axis represent cell groups and chromosomes respectively. Red gradient denotes gains and blue denotes loss.

Suppl. Figure 7

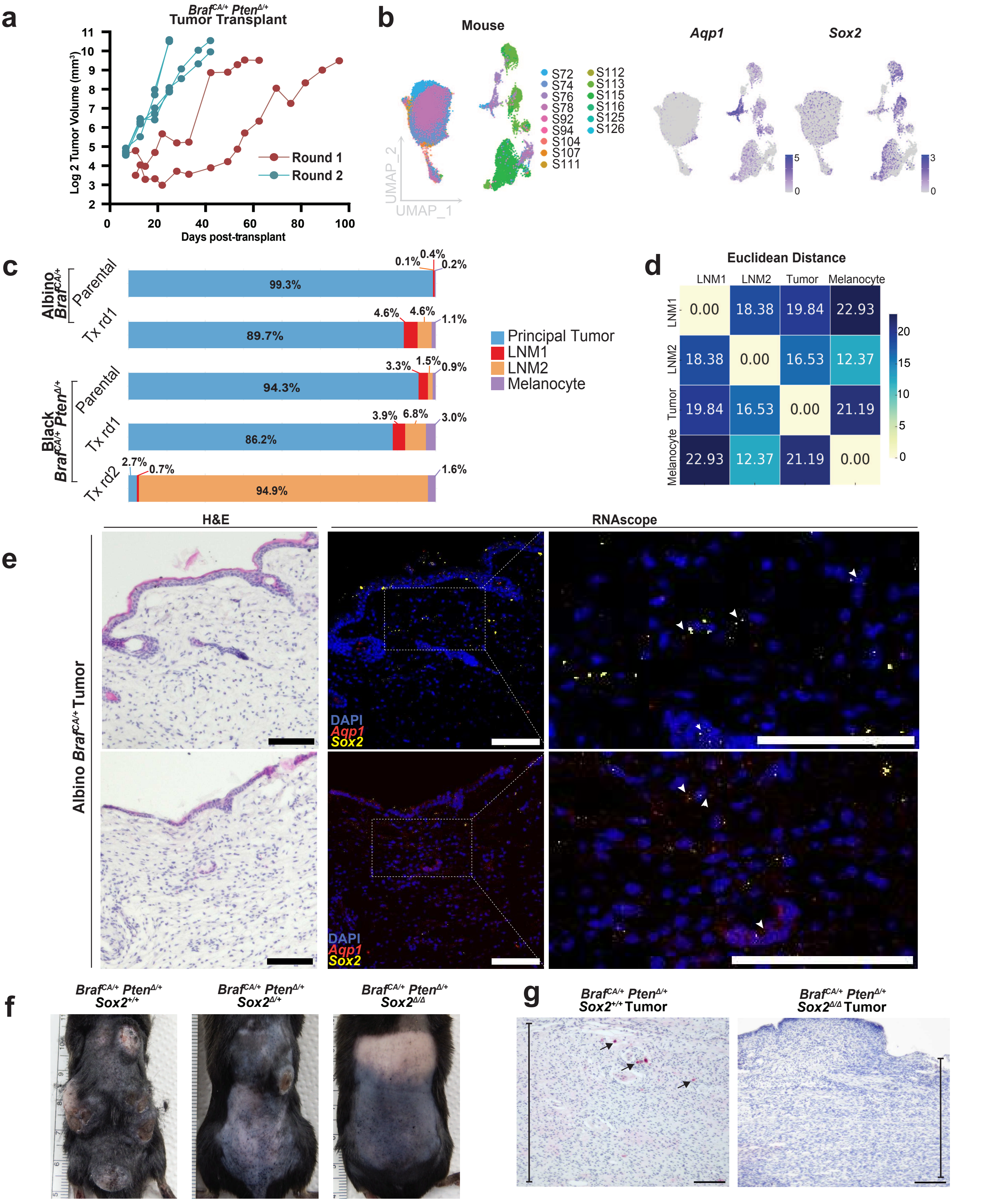

Supplementary Figure 7. A. Tumors were transplanted from one Black *Braf*$^{CA/+}$; *Pten*$^{\Delta/+}$ tumor into two NSG hosts and harvested (round 1). One tumor transplanted in the first round was harvested and re-transplanted into five NSG hosts (round 2). Growth curve for each tumor is recorded. Note the time delay for tumor initiation is shortened after re-transplantation. Source data are provided as a Source Data file. **B.** Additional UMAP visualizations of the NC-derived clusters in Fig 3B, with distribution labeled by mouse and feature plots highlighting LNM clusters labelled by *Aqp*1 and *Sox2*. Color bar denotes expression level. **C.** Cell proportion plots in NC-derived clusters across sample types from two sets of transplantation experiments. Note both LNM1 and LNM2 populations are present in all samples and persist through transplantation rounds. Tx: transplant. Rd: round of transplantation. **D.** Heatmap of Euclidean distances calculated between NC-derived clusters in Fig 3B using top 30 embeddings from Harmony space. **E.** RNAscope in-situ hybridization of *Aqp1* and *Sox2* in two representative Albino *Braf*$^{CA/+}$tumors of the three biological replicates that were stained. Co-expression is marked by white arrows. Corresponding H&E staining is shown. Scale bar: 100um. **F.** Representative mice of the indicated genotypes are shown. Note the inhibition of tumor when *Sox2* is eliminated by *Tyr::CreERT2*. **G.** Tumors of the indicated genotypes were stained with a Sox2 antibody (pink staining). Note the staining indicating *Sox2* deletion in the sole *Braf*$^{CA/+}$; *Pten*$^{\Delta/+}$ *Sox2*$^{\Delta/\Delta}$ mouse tumor we found in our cohort, compared to the positive staining (indicated by arrows) in a *Sox2*-wildtype tumor. Samples are counterstained with hematoxylin. Black brackets denote tumor-containing regions. Immunohistochemistry micrographs are representative images from staining performed on three independent biological replicates. Scale bar: 100um.

Suppl. Figure 8

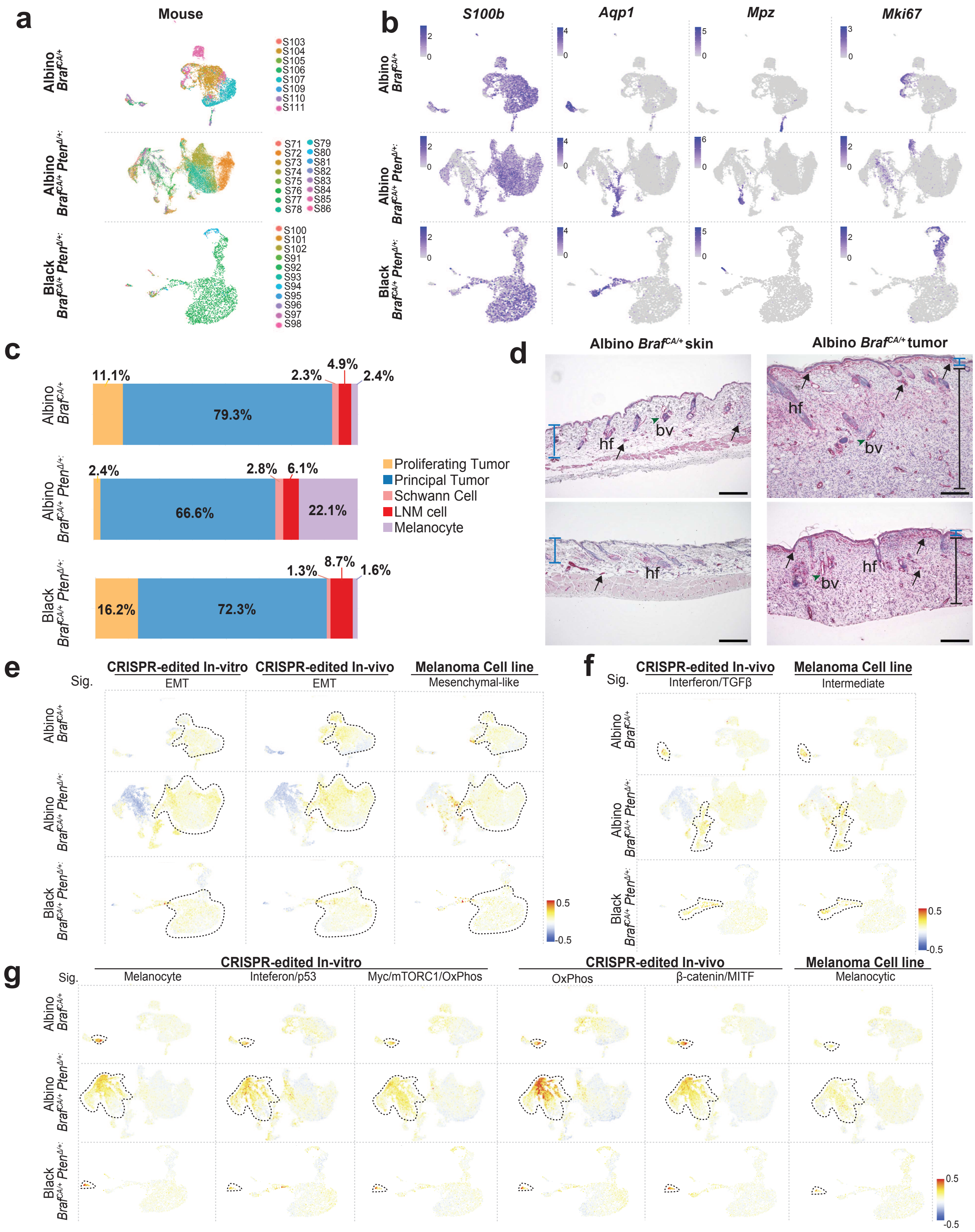

Supplementary Figure 8. A-B: UMAP visualization of single cell gene expression profiles by mouse and (B) feature plots of NC-derived clusters from three datasets in Fig 3A. Distinct cell-types are highlighted: Principal tumor cells (*S100b*), LNM cells (*Aqp1*), Schwann cells (*Mpz*), and a highly proliferative subset of principal tumor cells (*Mki67*). Color bar denotes expression level. **C.** Cell proportion plots of NC-derived clusters across three datasets in Fig 3A. **D.** Albino *Braf*$^{CA/+}$ skin and tumor samples were stained with a Aqp1 antibody to identify LNM cells (pink staining of Aqp1+; black arrows). LNM cells are found in both the superficial (blue brackets) and deeper dermis (black brackets). Representative micrographs of three independent biological replicates are shown. hf: hair follicles. bv: blood vessels (green arrows). Scale bar: 200um. **E-G.** Additional gene signature comparisons were performed comparing with published datasets[18, 19, 22, 55, 56] (Table S25). Gene signatures that aligned primarily with principal tumor cells, LNM cells, and melanocytes are shown in E-G respectively. Color bar denotes membership agreement level. Black dashes outline cell-types in the individual datasets in Fig 4A. EMT: Epithelial-mesenchymal transitions. Sig: signature. OxPhos: Oxidative phosphorylation.
