# Supplementary material for: Uncovering minimal pathways in melanoma initiation
Source: Nat Commun. 2025 Jun 26;16:5369. doi: 10.1038/s41467-025-60742-0 (PMC12202705; doi:10.1038/s41467-025-60742-0)
Supplement: Supplementary file 3 — Description of Additional Supplementary Files [file 41467_2025_60742_MOESM3_ESM.pdf]

## Description of Additional Supplementary Files

**Supplementary Data 1:** Albino *Braf*$^{CA/+}$ Tumor #1: annotation of all single nucleotide variants listed in Fig 1D

**Supplementary Data 2:** Albino *Braf*$^{CA/+}$ Tumor #1: annotation of all single nucleotide variants located in exons, listed in Fig 1D

**Supplementary Data 3:** Albino *Braf*$^{CA/+}$ Tumor #2: annotation of all single nucleotide variants listed in Fig 1D

**Supplementary Data 4:** Albino *Braf*$^{CA/+}$ Tumor #2: annotation of all single nucleotide variants located in exons, listed in Fig 1D

**Supplementary Data 5:** Albino *Braf*$^{CA/+}$ Tumor #3: annotation of all single nucleotide variants listed in Fig 1D

**Supplementary Data 6:** Albino *Braf*$^{CA/+}$ Tumor #3: annotation of all single nucleotide variants located in exons, listed in Fig 1D

**Supplementary Data 7:** Shared nonsynonymous single nucleotide variants among three albino *Braf*$^{CA/+}$ tumors listed in Fig 1D and ST2, 4, 6

**Supplementary Data 8:** "Flna mutation on exon 21 and 45 on skin samples with no Braf mutation (n=7), *Braf*$^{CA/+}$ and *Braf*$^{CA/+}$; *Pten*$^{\Delta/+}$ tumor samples (n=7), and tumor-adjacent skin samples (n=7)

**Supplementary Data 9:** Albino *Braf*$^{CA/+}$ Tumor #1: Structural variants in tumor, listed in Fig 1E, identified by the BreakDancer pipeline, with skin and spleen as control (tumor - skin - spleen)

**Supplementary Data 10:** Albino *Braf*$^{CA/+}$ Skin #1: Structural variants in skin, identified by the BreakDancer pipeline, with spleen as control (skin - spleen)

**Supplementary Data 11:** Albino *Braf*$^{CA/+}$ Tumor #1: Copy number variants, listed in Fig 1E, identified by the CNVkit pipeline

**Supplementary Data 12:** Albino *Braf*$^{CA/+}$ Tumor #2: Structural variants in tumor, listed in Fig 1E, identified by the BreakDancer pipeline, with skin and spleen as control (tumor - skin - spleen)

**Supplementary Data 13:** Albino *Braf*$^{CA/+}$ Skin #2: Structural variants in skin, identified by the BreakDancer pipeline, with spleen as control (skin - spleen)

**Supplementary Data 14:** Albino *Braf*$^{CA/+}$ Tumor #2:Copy number variants in tumor, listed in Fig 1E, identified by the CNVkit pipeline

**Supplementary Data 15:** Albino *Braf*$^{CA/+}$ Tumor #3: Structural variants in tumor, listed in Fig 1E, identified by the BreakDancer pipeline, with skin and spleen as control (tumor - skin - spleen)

**Supplementary Data 16:** Albino *Braf*$^{CA/+}$ Skin #3: Structural variants in skin, identified by the BreakDancer pipeline, with spleen as control (skin - spleen)

**Supplementary Data 17:** Albino *Braf*$^{CA/+}$ Tumor #3: Copy number variants, listed in Fig 1E, identified by the CNVkit pipeline

**Supplementary Data 18:** Metadata for all samples subjected to scRNA-seq, that were described in Fig 2A and Fig3A

**Supplementary Data 19:** Top 30 differentially expressed genes for each cell type that are depicted in the UMAP plot in Fig 2B

**Supplementary Data 20:** Top 30 differentially expressed genes for each cell type that are depicted in the UMAP plot in Fig 2C

**Supplementary Data 21:** Top 30 Differentially expressed genes for each cell type that are depicted in the UMAP plot in Fig 3D

**Supplementary Data 22:** Euclidean distances calculated between NC-derived cell clusters (35, 527 cells from Fig 2B) using the average embeddings from the top 10 Principal Components (PCs) (Fig S3F)

**Supplementary Data 23:** Euclidean distances calculated between NC-derived clusters (22,732 cells from transplanted and parental tumors in Fig 3B) using the average embeddings from the top 30 Harmony embeddings (Fig S6D)

**Supplementary Data 24:** Gene signature sets used for determining the comparative "membership scores" in Fig 4

**Supplementary Data 25:** "Additional gene signature sets used to determine "membership scores" depicted in Fig S7E-G

**Supplementary Data 26:** Differentially expressed gene (DEG) lists from RNA velocity analysis of three individual tumors using the software scVelo, shown in Fig 5B-D

**Supplementary Data 27:** Differentially expressed gene (DEG) list from Mutrans transitional analysis of three tumors, shown in Fig 5E
