# Supplementary material for: Uncovering minimal pathways in melanoma initiation
Source: Nat Commun. 2025 Jun 26;16:5369. doi: 10.1038/s41467-025-60742-0 (PMC12202705; doi:10.1038/s41467-025-60742-0)
Supplement: Supplementary file 5 — Reporting Summary [file 41467_2025_60742_MOESM5_ESM.pdf]

Corresponding author(s): Anand K Ganesan

Last updated by author(s): 05/13/2025

Reporting Summary

Nature Portfolio wishes to improve the reproducibility of the work that we publish. This form provides structure for consistency and transparency in reporting. For further information on Nature Portfolio policies, see our Editorial Policies and the Editorial Policy Checklist.

Statistics

For all statistical analyses, confirm that the following items are present in the figure legend, table legend, main text, or Methods section.

n/a	Confirmed	
☐	☒	The exact sample size (*n*) for each experimental group/condition, given as a discrete number and unit of measurement
☐	☒	A statement on whether measurements were taken from distinct samples or whether the same sample was measured repeatedly
☐	☒	The statistical test(s) used AND whether they are one- or two-sided<br>*Only common tests should be described solely by name; describe more complex techniques in the Methods section.*
☒	☐	A description of all covariates tested
☒	☐	A description of any assumptions or corrections, such as tests of normality and adjustment for multiple comparisons
☐	☒	A full description of the statistical parameters including central tendency (e.g. means) or other basic estimates (e.g. regression coefficient) AND variation (e.g. standard deviation) or associated estimates of uncertainty (e.g. confidence intervals)
☐	☒	For null hypothesis testing, the test statistic (e.g. *F*, *t*, *r*) with confidence intervals, effect sizes, degrees of freedom and *P* value noted<br>*Give P values as exact values whenever suitable.*
☒	☐	For Bayesian analysis, information on the choice of priors and Markov chain Monte Carlo settings
☒	☐	For hierarchical and complex designs, identification of the appropriate level for tests and full reporting of outcomes
☐	☒	Estimates of effect sizes (e.g. Cohen's *d*, Pearson's *r*), indicating how they were calculated

Our web collection on statistics for biologists contains articles on many of the points above.

Software and code

Policy information about availability of computer code

Data collection	Illumina NovaSeq 6000 was used for whole-genome sequencing.<br>Illumina HiSeq 4000 was used for single-cell RNA sequencing.
Data analysis	FastQC 0.11.9, Trimmomatic 0.39, BreakDancer 1.1.2, SigProfiler, CNVkit 0.9.11, Annovar(version date 2020-06-07) were used to analyze Whole Genome Sequencing data.<br>Cellranger 3.1.0, Seurat 3.1.5, scVelo 0.1.23, MuTrans, Harmony 1.2.0, and inferCNV 1.20.0 were used to analyze single-cell RNA sequencing data.<br>ImageJ 1.53q, Graphpad prism 10.0.0 were used for image analysis and growth curve graphing.

For manuscripts utilizing custom algorithms or software that are central to the research but not yet described in published literature, software must be made available to editors and reviewers. We strongly encourage code deposition in a community repository (e.g. GitHub). See the Nature Portfolio guidelines for submitting code & software for further information.

Data

Policy information about availability of data

All manuscripts must include a data availability statement. This statement should provide the following information, where applicable:

- Accession codes, unique identifiers, or web links for publicly available datasets
- A description of any restrictions on data availability
- For clinical datasets or third party data, please ensure that the statement adheres to our policy

The scRNA-sequencing and WGS sequencing data generated in this study have been deposited in the SRA repository under BioProject accession code PRJNA1172319 [https://www.ncbi.nlm.nih.gov/bioproject/PRJNA1172319] and in the GEO repository under accession code GSE279468 [https://www.ncbi.nlm.nih.gov/geo/query/acc.cgi?acc=GSE279468]. Source data are provided with this paper. Processed human scRNA-seq data from previously published study Pozniak et al., Cell (2024) is publicly available at KU Leuven RDR [https://doi.org/10.48804/GSAXBN].

Research involving human participants, their data, or biological material

Policy information about studies with human participants or human data. See also policy information about sex, gender (identity/presentation), and sexual orientation and race, ethnicity and racism.

Reporting on sex and gender	N/A
Reporting on race, ethnicity, or other socially relevant groupings	N/A
Population characteristics	N/A
Recruitment	N/A
Ethics oversight	N/A

Note that full information on the approval of the study protocol must also be provided in the manuscript.

Field-specific reporting

Please select the one below that is the best fit for your research. If you are not sure, read the appropriate sections before making your selection.

☒ Life sciences ☐ Behavioural & social sciences ☐ Ecological, evolutionary & environmental sciences

For a reference copy of the document with all sections, see nature.com/documents/nr-reporting-summary-flat.pdf

Life sciences study design

All studies must disclose on these points even when the disclosure is negative.

Sample size	No statistical methods were used to pre-determine the sample size. A total of 128 animals were used, including black wildtype mice (n=8), black BrafCA/+ mice (n=10), black BrafCA/+ PtenΔ/+ mice (n=25), albino wildtype mice (n=5), albino PtenΔ/+ mice (n=2), albino BrafCA/+ mice (n=33), albino BrafCA/+ PtenΔ/+ mice (n=18), round 1 tumor transplanted NSG mice (n=4), round 2 transplanted NSG mice (n=5), black BrafCA/+,PtenΔ/+ Sox2+/+ mice (n=5), black Sox2 BrafCA/+,PtenΔ/+ Sox2Δ/+ mice (n=9), and black BrafCA/+,PtenΔ/+ Sox2Δ/Δ mice (n=4) were used.
Data exclusions	No data was excluded from the analyses
Replication	Experiments were performed for at least 2 biological replicates for each group.
Randomization	There was no randomization of the samples.
Blinding	Investigators were not blinded to the samples.

Reporting for specific materials, systems and methods

We require information from authors about some types of materials, experimental systems and methods used in many studies. Here, indicate whether each material, system or method listed is relevant to your study. If you are not sure if a list item applies to your research, read the appropriate section before selecting a response.

Materials & experimental systems

n/a	Involved in the study
☐	☒ Antibodies
☒	☐ Eukaryotic cell lines
☒	☐ Palaeontology and archaeology
☐	☒ Animals and other organisms
☒	☐ Clinical data
☒	☐ Dual use research of concern
☒	☐ Plants

Methods

n/a	Involved in the study
☒	☐ ChIP-seq
☒	☐ Flow cytometry
☒	☐ MRI-based neuroimaging

Antibodies

Antibodies used

anti-Aqp1 rabbit antibody (EMD Millipore AB2219) was used for immunohistochemistry.
anti-Sox2 rabbit antibody (Abcam Ab97959) was used for immunohistochemistry
anti-Pten antibody (Cell Signaling 9559S) was used for immunofluoresence primary staining
Alexa Fluor 594 goat anti-Rabbit IgG antibody (Life Technologies A11037) was used for immunofluoresence secondary staining

Validation

anti-Aqp1 rabbit antibody - validated applications: WB, IHC; validated reactivity: mouse, rat, human
anti-Sox2 rabbit antibody - validated applications: IHC-P, WB, ICC/IF; validated reactivity: mouse, rat, human
anti-Pten rabbit antibody - validated applications: IHC, WB, IP; validated reactivity: house, rat, human, monkey
Alexa Fluor 594 goat anti-Rabbit IgG antibody - validated applications: ICC/IF, Flow; validated reactivity: rabbit

Animals and other research organisms

Policy information about studies involving animals; ARRIVE guidelines recommended for reporting animal research, and Sex and Gender in Research

Laboratory animals

BrafCA/+;Tyr::CreER(C56BL/6), BrafCA/+;Ptenfl/+;Tyr::CreER(C56BL/6), NSG, C56BL/6 mice.

Wild animals

no wild animals were used.

Reporting on sex

65 males and 62 females were used in the study. Gender information from 1 wild-type mice from post-natal day 50 was not collected.

Field-collected samples

no field-collected samples.

Ethics oversight

This study was performed in strict accordance with the recommendation from University Laboratory Animal Resources (ULAR). All the animals were handled according to approved institutional animal care and use committee (IACUC) protocol (AUP17-230, AUP-20-161, AUP-23-116) at University of California Irvine.

Note that full information on the approval of the study protocol must also be provided in the manuscript.

Plants

Seed stocks

Report on the source of all seed stocks or other plant material used. If applicable, state the seed stock centre and catalogue number. If plant specimens were collected from the field, describe the collection location, date and sampling procedures.

Novel plant genotypes

Describe the methods by which all novel plant genotypes were produced. This includes those generated by transgenic approaches, gene editing, chemical/radiation-based mutagenesis and hybridization. For transgenic lines, describe the transformation method, the number of independent lines analyzed and the generation upon which experiments were performed. For gene-edited lines, describe the editor used, the endogenous sequence targeted for editing, the targeting guide RNA sequence (if applicable) and how the editor was applied.

Authentication

Describe any authentication procedures for each seed stock used or novel genotype generated. Describe any experiments used to assess the effect of a mutation and, where applicable, how potential secondary effects (e.g. second site T-DNA insertions, mosiacism, off-target gene editing) were examined.
